# The HIV care cascade: Japanese perspectives

**DOI:** 10.1371/journal.pone.0174360

**Published:** 2017-03-20

**Authors:** Aikichi Iwamoto, Rikizo Taira, Yoshiyuki Yokomaku, Tomohiko Koibuchi, Mahbubur Rahman, Yoko Izumi, Kenji Tadokoro

**Affiliations:** 1 Japan Agency for Medical Research and Development, Tokyo, Japan; 2 Blood Service Headquarters, Japanese Red Cross Society, Tokyo, Japan; 3 Clinical Research Center, Nagoya Medical Center, National Hospital Organization. Nagoya, Japan; 4 The Institute of Medical Science, the University of Tokyo, Tokyo, Japan; 5 St. Luke’s International University, Tokyo, Japan; Vanderbilt University, UNITED STATES

## Abstract

Japan has been known as a low HIV-prevalence country with a concentrated epidemic among high-risk groups. However, it has not been determined whether Japan meets the 90-90-90 goals set by the Joint United Nations Programme on HIV/AIDS (UNAIDS)/World Health Organization (WHO). Moreover, to date, the HIV care cascade has not been examined. We estimated the total number of diagnosed people living with HIV/AIDS (PLWHA) (n = 22,840) based on legal reports to the Ministry of Health, Labour and Welfare by subtracting the number of foreigners who left Japan (n = 2,273) and deaths (n = 2,321) from the cumulative diagnosis report (n = 27,434). The number of total undiagnosed PLWHA was estimated by age and sex specific HIV-positive rates observed among first-time blood donors between 2011–2015 in Japan. Our estimates show that 14.4% (n = 3,830) of all PLWHA (n = 26,670) were undiagnosed in Japan at the end of 2015. The number of patients retained in care (n = 20,615: 77.3% of PLWHA), the percentage of those on antiretroviral therapy (n = 18,921: 70.9% of PLWHA) and those with suppressed viral loads (<200 copies/mL; n = 18,756: 70.3% of PLWHA) were obtained through a questionnaire survey conducted in the AIDS Core Hospitals throughout the country. According to these estimates, Japan failed to achieve the first two of the three UNAIDS/WHO targets (22,840/26,670 = 85.6% of HIV-positive cases were diagnosed; 18,921/22,840 = 82.8% of those diagnosed were treated; 18,756/18,921 = 99.1% of those treated experienced viral suppression). Although the antiretroviral treatment uptake and success after retention in medical care appears to be excellent in Japan, there are unmet needs, mainly at the surveillance level before patients are retained in care. The promotion of HIV testing and treatment programs among the key affected populations (especially men who have sex with men) may contribute to further decreasing the HIV epidemic and achieving the UNAIDS/WHO targets in Japan.

## Introduction

The introduction and improvement of antiretroviral treatment (ART) decreased the occurrence of not only AIDS events [1,2] but also non-AIDS events [3,4], thereby contributing tremendously to improvements in the life expectancy of people living with HIV/AIDS (PLWHA) [5,6]. In addition to the benefits observed in individual PLWHA, ART greatly reduces HIV transmission in stable, serodiscordant couples, supporting the notion of Treatment as Prevention (TasP) [7]. While observational studies reported favorable results regarding the population-level effects of ART on HIV transmission [8–11] and a mathematical model suggested its possible elimination through universal testing and treatment [12], the impact of ART on the HIV incidence at the population level remains controversial [13,14]. It has been reported that TasP has a potentially higher likelihood to succeed in resource-rich countries with concentrated HIV epidemics [15], but the use of ART nationwide ART to effectively prevent HIV is a formidable task. A substantial loss of eligible people has been observed from stage to stage in HIV care even in resource-rich countries. For example, studies conducted in the United States showed that 20% of PLWHA were unaware of their status, and more than 50% of those diagnosed with HIV were not retained in HIV medical care [16,17].

Japan has been known as a low-prevalence country with a concentrated epidemic among men who have sex with men (MSM) [18]. While Japan has a good system of reporting infectious diseases and excellent national healthcare systems, there is a dearth of studies that comprehensively evaluate HIV care at the national level. Estimating unidentified PLWHA is a prerequisite for addressing the HIV prevalence and HIV care cascade in Japan.

Voluntary and unpaid blood donations across the country were introduced in 1982 by the Japanese Red Cross Society (JRCS) in cooperation with the national and local governments [19]. Approximately 5 million blood donations have been recorded annually in recent years. Blood donation is open to people aged 16 to 69 years, which is the age group constituting a great majority of PLWHA in this country [20]. To estimate the total undiagnosed HIV-positive population living in Japan, we extrapolated the age and sex specific HIV-positive rates observed in first-time voluntary blood donors to the complete population of Japan. By combining this estimate of undiagnosed PLWHA with the PLWHA identified by the existing surveillance system, we assessed the total number of PLWHA. The numbers of patients retained in care, those on ART and those successfully treated were obtained by a nationwide questionnaire survey conducted in the AIDS Core Hospitals. In this study, we aimed to estimate the current HIV care cascade in Japan across the continuum of care along with the goals set by the Joint United Nations Programme on HIV/AIDS (UNAIDS)/World Health Organization (WHO) (90-90-90) [21].

## Materials and methods

### Ethics statement

The questionnaire survey conducted in this study was approved by the Internal Review Board of the Nagoya Medical Center (2016–86).

### Estimation of the number of undiagnosed PLWHA

All blood units donated to the JRCS were routinely screened for anti-HIV antibodies and HIV-specific nucleotides. The HIV prevalence data among people who donated blood for the first time between 2011–2015 were weighted by age and sex distribution of the population in Japan as of October 1, 2015 [22].

### HIV surveillance data in Japan

Cumulative numbers of reported HIV and AIDS cases and AIDS-related death were obtained from the Annual AIDS Surveillance Report compiled by the National AIDS Surveillance Committee [20]. The HIV/AIDS reporting system was implemented in 1985 in Japan. HIV infection and AIDS have been designated notifiable infectious diseases under the “AIDS Prevention Law” since 1989 and the “Act on Prevention of Infectious Diseases and Medical Care for Patients Suffering Infectious Diseases (Infectious Disease Law)” since 1999. At the first diagnosis, HIV-positive patients without an AIDS-associated disease are reported as an HIV-infection while AIDS cases are reported as AIDS to the Ministry of Health Labour and Welfare (MHLW). Municipal healthcare centers and related facilities, where free anonymous testing is provided, report approximately 45% of new HIV infections per year [23]. Positive cases are referred from municipal healthcare centers to AIDS Core Hospitals designated by MHLW where ART is provided according to the guidelines. However, 55% of new HIV infections and essentially all new AIDS cases are reported by the physicians who diagnose them in hospitals and clinics. As reporting HIV/AIDS cases is mandatory by law, this system is supposedly highly compliant in Japan. According to the law, healthcare providers must report cases of HIV infections without AIDS-associated disease(s) as HIV infections or cases of HIV infections with AIDS-associated disease(s) as AIDS cases that have not been previously reported [24]. Healthcare providers should also report any AIDS or death case separately as a medical condition change (MCC-AIDS or MCC-death) if the case had been previously reported as an HIV infection or AIDS. While MCC-deaths are mandatory to report by the “AIDS Prevention Law”, MCC has been designated an arbitrary report in the “Infectious Disease Law” since 1999. Thus, the total number of MCC-deaths reported is likely to be an underestimation of the actual AIDS-related deaths. To address this uncertainty, we used a database containing information on the leading cause of death based on vital statistics [25]. In addition, we also used data related to AIDS-related deaths obtained from the hospital survey we conducted.

Hemophiliacs and patients with acquired coagulation disorders who were infected via contaminated blood coagulation factors (CBCF) have been registered through a Nationwide Survey on Coagulation Disorders [26]. Since these data had been outside the purview of the AIDS-related law, we incorporated the numbers of HIV infections and AIDS-related deaths using this database. Then, based on the cumulative report (diagnoses), HIV-positive foreigners, mortality and undiagnosed PLWHA, we calculated the “% diagnosed patients”.

### Nationwide questionnaire survey of the AIDS Core Hospitals

Three hundred and eighty-two hospitals across the country have been designated AIDS Core Hospitals by the MHLW. To obtain the maximum response, the survey questionnaires were sent through the Health Service Bureau of the 47 municipalities of Japan that supervise the hospitals. This questionnaire included items regarding (1) annual new visits, (2) regular visits, (3) number of the patients on ART, (4) treatment failure, and (5) cumulative death (S1 Questionnaire). The study period was from January 1, 2015 to December 31, 2015. We defined the sum of in- and out-patients with the diagnosis of HIV infection or AIDS within three months (from October 1, 2015 to December 31, 2015) as regular visitors, i.e., patients "retained in care". The success or failure of ART was determined by the attending physicians 6 months after the treatment was initiated. The treatment was determined to be a failure if the viral load (VL) was more than 200 copies/mL on two successive occasions.

### Longitudinal survey of the ART coverage in an AIDS Core Hospital

A hospital, affiliated with the Institute of Medical Science, the University of Tokyo (IMSUT Hospital), stated the care for PLWHA in 1986. One of the authors (AI) was also in charge of the HIV Clinic in the IMSUT Hospital from January 1995 –March 2015. Longitudinal ART coverage since 1998, when the triple combination therapy had become widely available in Japan, was investigated based on the survey data collected from the IMSUT Hospital.

### Statistical analysis

We used chi-square tests to compare categorical variables. P-values <0.05 were considered statistically significant.

### Sensitivity analyses

Although our results are based on the best available estimates, we conducted sensitivity analyses to examine the effects of changes in the various parameters we used in our calculations. We examined the effect of 10–20% changes in HIV detection based on blood donation to calculate its effect on overall undiagnosed PLWHA and % diagnosed patients. We also examined the effect of 5–20% changes of double counting, foreigners who left Japan and cumulative death on % diagnosed patients.

## Results

### Estimated number of undiagnosed PLWHA in Japan

Overall, 118 HIV-positive cases (0.005%) were identified based on first-time blood donors (n = 2,283,450) between 2011–2015 (Table 1). The male/female (114/4) ratio was similar to that of newly reported HIV/AIDS cases identified through the Annual AIDS Surveillance during the same period (7,165/383) (P = 0.527) [20]. More HIV-positive cases were observed among males in the 25–49 age bracket compared to any other age category, which is similar to the national surveillance data [20]. Since the sample size was small, we used the mean value of all HIV-positive infections identified over 5 years (2011–2015). By extrapolating using the published national age- and sex-matched overall population, we estimated that 3,830 PLWHA were undiagnosed in Japan in 2015.

**Table 1:** HIV-positive cases in first-time blood donors.

Age	Sex		Year					Mean	National age and sex specific population in 2015	Age and sex specific HIV+ people
			2011	2012	2013	2014	2015			
16–19	M	HIV+	2	1	0	2	0	1	2,470,000	31
		Donors	79,844	83,187	84,929	75,221	74,240	79,240		
	F	HIV+	0	1	0	0	0	0.2	2,343,000	8
		Donors	60,224	60,129	63,377	50,852	45,022	55,921		
20–24	M	HIV+	9	0	4	3	1	3.4	3,046,000	151
		Donors	74,511	73,503	72,565	64,363	58,681	68,725		
	F	HIV+	1	0	0	0	0	0.2	2,922,000	13
		Donors	51,575	46,828	47,573	39,243	32,238	43,491		
25–29	M	HIV+	9	3	7	5	7	6.2	3,256,000	560
		Donors	40,907	38,805	37,493	32,410	30,537	36,030		
	F	HIV+	1	1	0	0	0	0.4	3,154,000	69
		Donors	23,270	20,125	19,873	15,569	12,571	18,282		
30–34	M	HIV+	9	0	5	3	2	3.8	3,685,000	596
		Donors	28,457	25,372	23,754	20,465	19,501	23,510		
	F	HIV+	0	0	0	0	0	0	3,606,000	0
		Donors	15,361	12,883	12,211	9,501	7,824	11,556		
35–39	M	HIV+	4	2	1	2	3	2.4	4,204,000	485
		Donors	27,200	22,478	20,239	17,700	16,409	20,805		
	F	HIV+	0	0	0	0	0	0	4,112,000	0
		Donors	15,434	12,203	10,483	8,059	6,733	10,582		
40–44	M	HIV+	3	4	4	5	1	3.4	4,914,000	885
		Donors	20,905	18,706	17,321	18,080	19,430	18,888		
	F	HIV+	0	0	0	0	0	0	4,818,000	0
		Donors	13,713	11,629	10,365	9,426	9,067	10,840		
45–49	M	HIV+	1	3	1	2	1	1.6	4,355,000	527
		Donors	13,045	11,853	11,038	13,512	16,715	13,233		
	F	HIV+	0	0	0	0	0	0	4,308,000	0
		Donors	9,847	8,881	7,858	8,387	9,128	8,820		
50–54	M	HIV+	2	0	0	0	1	0.6	3,968,000	259
		Donors	8,579	7,573	7,082	9,523	13,180	9,187		
	F	HIV+	0	0	0	0	0	0	3,962,000	0
		Donors	8,766	7,834	7,042	7,694	8,919	8,051		
55–59	M	HIV+	0	1	1	0	0	0.4	3,730,000	246
		Donors	5,898	4,908	4,552	6,168	8,832	6,072		
	F	HIV+	0	0	0	0	0	0	3,786,000	0
		Donors	6,054	5,410	4,767	4,998	5,972	5,440		
60–64	M	HIV+	0	0	0	0	0	0	4,151,000	0
		Donors	4,127	3,079	2,889	3,941	5,591	3,925		
	F	HIV+	0	0	0	0	0	0	4,304,000	0
		Donors	4,049	3,347	3,012	2,914	3,336	3,332		
65–69	M	HIV+	0	0	0	0	0	0	4,660,000	0
		Donors	225	237	228	272	472	287		
	F	HIV+	0	0	0	0	0	0	4,984,000	0
		Donors	201	232	209	220	280	228		
Total HIV+	M	HIV+	39	14	23	22	16	22.8	Total HIV+	3,830
	F	HIV+	2	2	0	0	0	0.8		
Total donors			512,192	479,202	468,860	418,518	404,678			

Total first-time blood donors between 2011–2015: 2,283,450

Total HIV-positive between 2011–2015: 118

M: Male, F: Female

Age and sex specific HIV+ people = (Mean HIV+) / (Mean first-time donors) x (National age and sex specific population in 2015)

### Estimation of % diagnosed PLWHA in Japan

**Fig 1:**
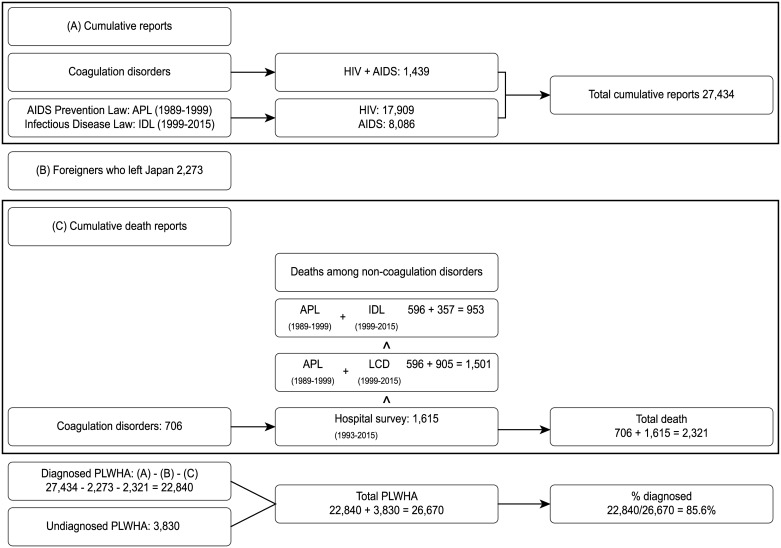
Estimation of diagnosed PLWHA as of December 31 2015. (A)Cumulative diagnosis reports were collected from different data sources. (B)The number of foreigners who left Japan was estimated as written in “Materials and Methods” based on a previous report [27]. (C)Three different sources of deaths were available for non-coagulation disorders. The highest number (Hospital survey) was chosen to estimate the cumulative death number. % diagnosed was calculated based on the data in (A), (B), (C) and the number of undiagnosed PLWHA (Table 1). APL, AIDS Prevention Law; IDL, Infectious Disease Law; LCD, Leading Cause of Death.

We calculated the number of diagnosed PLWHA by subtracting the number of registered foreigners who left Japan and the mortality data from the cumulative number of diagnosis/reports based on non-overlapping databases. In total, 27,434 HIV-positive cases were diagnosed in Japan by December 31, 2015 (Fig 1). Among the 27,434 HIV-positive cases, 4,217 (2,955 HIV infection and 1,262 AIDS cases) were foreigners. Nakao et al. estimated that among the 3,941 HIV-positive foreigners, 1,817 (46.1%) stayed in Japan, and 53.9% left the country by the year 2013 [27]. We assumed that among the 4,217 foreigners, 53.9% (n = 2,273) left Japan, and subtracted them from the reported number (Fig 1).

Mortality related to CBCF has been investigated separately, and we observed that 706 deaths had been registered by May 31, 2015 (the latest estimate at the time of the writing of this manuscript). Cumulatively, 596 or 357 MCC-deaths were reported in accordance with the “AIDS Prevention Law” between 1985–1999 or the “Infectious Disease Law” between 1999–2015, respectively. There were 1,659 (706+596+357) total deaths, including CBCF-related deaths (Fig 1). AIDS-related deaths reported in the list of leading causes of death by “Vital statistics” (n = 905) between 1999–2015 were higher than those in the report obtained through the “Infectious Disease Law” (n = 357). There were 2,207 (706+596+905) total deaths, including CBCF-related deaths. Cumulative deaths of non-hemophiliac HIV-infected patients reported by the survey of AIDS Core Hospitals totaled 1,615. In this calculation, total AIDS-related deaths in Japan numbered 2,321 (706+1615). We considered AIDS-related death information reported from the core hospitals survey as the most reliable estimate. Therefore, we estimated that 85.6% (22,840 = 27,434–2,273–2,321) of PLWHA (26,670 = 22,840 + 3,830) were diagnosed in Japan in 2015.

### HIV care cascade in Japan

HIV self-testing has not been approved in Japan to date. Approximately 45% of diagnoses and reporting have been performed in Municipal healthcare centers that provide free anonymous voluntary testing. Approximately 55% of HIV infection and AIDS cases have been reported by the attending physicians working in hospitals and clinics. Thus, essentially 100% of HIV-positive cases are diagnosed in the medical care system and are supposedly reported and linked to care efficiently in Japan.

**Fig 2:**
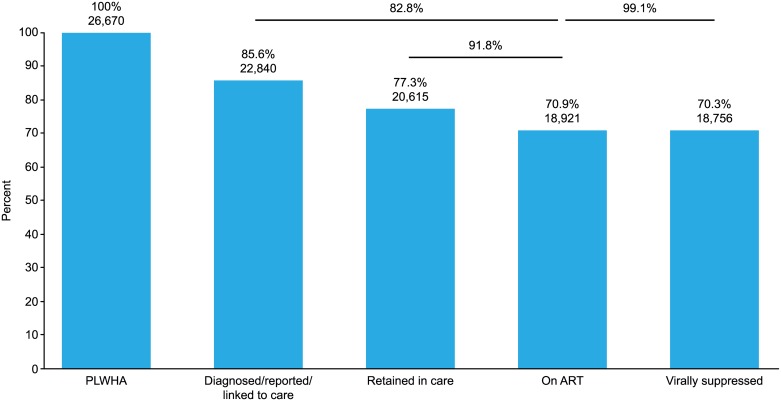
HIV care cascade in Japan. Columns in blue and the numbers and percentages above them show the care cascade using the number of PLWHA (26,670) as the denominator. The lines and percentages above them show the cascade calculated between the columns indicated by the lines.

Three hundred and fifty-six hospitals (93.2%) of the 382 AIDS Core Hospitals recorded the regular visits of a total of 21,228 patients in 2015 (Table 2). Three hundred and eight hospitals (80.6%), including almost all the major HIV care centers, responded with all the requested numbers listed on the survey questionnaire. Of the 20,615 patients who regularly visited a hospital (retained in care), 18,921 patients (91.8%) were on ART, and 18,756 of those (99.1%) were successfully treated. Data obtained from the AIDS surveillance and surveys conducted in the AIDS Core Hospitals calculated that 77.3%, 70.9% and 70.3% of PLWHA were retained in care, on ART and successfully treated in Japan, respectively (Fig 2). Based on the Joint UNAIDS/WHO 90-90-90 goals, our estimates showed that Japan failed to achieve the first two of the three goals (22,840/26,670 = 85.6% of HIV-positive patients were diagnosed; 18,921/22,840 = 82.8% of those diagnosed were treated; 18,756/18,921 = 99.1% of those treated experienced viral suppression).

**Table 2:** Results of the questionnaire survey in AIDS Core Hospitals.

AIDS Core Hospitals		Patients		
		Retained in care	On ART	Virally suppressed
Total	382			
Responded with number of patients	356	21,228		
Responded to all queries	308	20,615	18,921 (91.8%*)	18,756 (99.1%**)

*% On ART/retained in care. The sum of in- and out-patients with the diagnosis of HIV infection or AIDS within three months (from October 1 2015 to December 31 2015) were counted as "retained in care".

**% Virally suppressed/on ART

### Recent trend of HIV epidemic and ART scale-up in Japan

**Fig 3:**
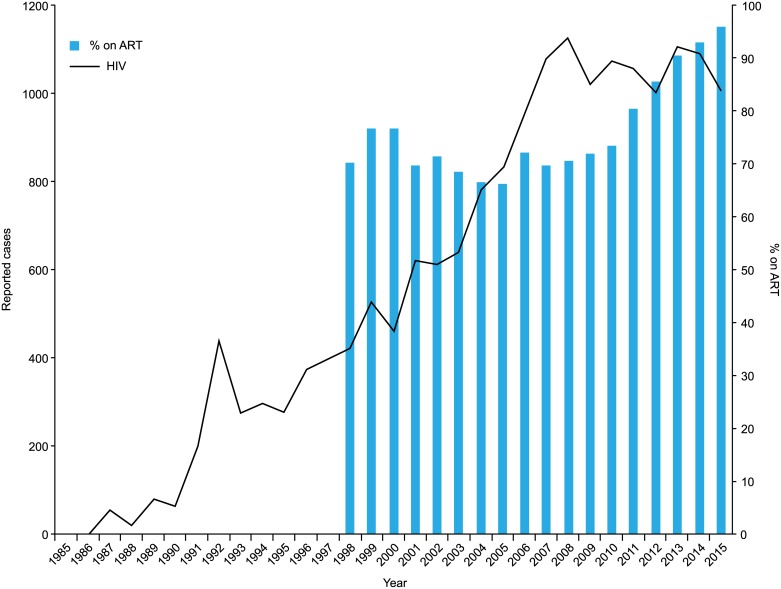
Annually reported new HIV infections and % on ART in an AIDS Core Hospital. Polygonal lines in black show the annually reported new HIV infections in Japan. Columns in blue show the percent of patients on ART in the IMSUT Hospital.

The number of newly reported HIV infections has soared from the beginning of the surveillance until 2008 (Fig 3) [20]. However, the number has plateaued since 2009 at the level of 1000–1,100 cases/year. Other related data, such as the rate of HIV seropositivity in donated blood [28] and physician visits with newly diagnosed HIV-infected cases in the major AIDS Core Hospitals (personal communications), also showed similar patterns (decreasing or plateauing since 2009). Thus, it is apparent that the actual incidence of HIV infections in Japan has decreased or plateaued since 2009.

Our survey based in AIDS Core Hospitals showed that 91.8% of HIV/AIDS patients retained in care were on ART in Japan. One medium size AIDS Core Hospital (IMSUT Hospital) had 516 of 538 (95.9%) patients on ART in December 2015. The viral load was < 20 copies /mL in 94.2% patients (486/516) and < 400 copies in 99.2% patients (512/516). Trends over this period of time showed that the percentage of patients on ART started exceeding 80% in 2011 and increased to ≥ 90% after 2013.

### Sensitivity analysis

To examine the effect of 10–20% changes in donors’ HIV-positive incidence on the % of diagnosed patients, we conducted a sensitivity analysis. Ten percent (2.4) or 20% (4.8) of mean HIV-positive cases/year (23.6) were weighted evenly to the four age- and sex-groups with the highest HIV-positive cases (S1 Table). The % of diagnosed patients changed by only 1–2% according to the 10–20% increase or decrease in the donors’ HIV-positive incidence.

We also conducted a sensitivity analysis to examine the influence of double counts, the number of foreigners and mortality on the % diagnosed (S2 Table). While the 5–10% decrease in the cumulative reports due to double counts changed the % diagnosed patients from 85.6% to 84.0–84.9%, the influence of the 10% change in the number of foreigners or mortality on the % diagnosed patients was only 0.1%.

## Discussion

We used universal blood donation records to estimate the undiagnosed HIV-positive population, while national surveillance data and nationwide questionnaires were utilized to define the HIV care continuum in Japan.

We estimated that 85.6% (22,840/26,670) of PLWHA are diagnosed in Japan. Although this rate is similar to the rates reported in Denmark, the United States and France [29], it indicated that Japan failed to achieve the first of the three UNAIDS/WHO 90-90-90 targets. Our estimation of undiagnosed PLWHA based on the HIV incidence in ambulant and healthy donors may have excluded undiagnosed PLWHA with a more progressed disease phase. In actuality, our estimate of undiagnosed PLWHA in Japan is lower than that previously reported based on a back-calculation method [30–32]. Since blood donation is a voluntary act and not a random sampling, our estimate of undiagnosed PLWHA may be biased downward if the campaign and pre-donation questionnaire were effective at excluding high-risk people. However, the result may be biased upward if high-risk donors are overrepresented despite the JRCS’s campaign for safer blood donations. However, the sensitivity analysis based on the variation (10–20%) in donors’ HIV-positive incidence did not change the estimate of % diagnosed patients substantially.

Japan also failed to achieve the second of the three UNAIDS/WHO 90-90-90 targets, which was 82.8% (% on ART/diagnosed: 18,921/22,840). The number of diagnosed PLWHA is a key number for the first two of the three targets. In Japan, free anonymous testing conducted in municipal healthcare centers identifies 45% of new HIV infections [23]. These centers report to the MHLW and link newly identified HIV/AIDS patients to a nearby AIDS Core Hospital. The rest of the infections and AIDS cases are diagnosed and reported through paid voluntary testing or during usual medical practice in clinics and hospitals. Physicians who diagnose an HIV infection or an AIDS case report to the MHLW via a municipal healthcare center. Although the passive surveillance system has been considered efficient in Japan, it has some limitations as well. First, the anonymous reporting system does not preclude double reporting. Double counting may occur when an HIV-positive client is tested in multiple healthcare centers or both the center and the referring physician report the same individual independently and anonymously. Double counting may have occurred, especially in the early days of HIV surveillance. Second, there is no efficient system to track HIV-positive foreigners. Fifteen percent (n = 4,217) of the cumulative reported HIV/AIDS cases (27,434) were identified as foreigners. We excluded the number of foreigners who might have left Japan after the diagnosis from our calculation of PLWHA as suggested by a recent report [27]. Third, AIDS mortality surveillance in Japan is weak, and mortality due to non-AIDS-related diseases has been increasing [33,34]. We observed that the cumulative AIDS mortality reported by AIDS Core Hospitals was higher than that reported by two government statistical reports. To address this inconsistency, we incorporated the highest observed value to generate a less biased estimate of the number of PLWHA. More precise mortality data of HIV-infected people should be incorporated in the national reporting system. However, the sensitivity analysis based on the variations in double counts, number of foreigners and mortality showed minimal changes in the overall estimates.

In addition, we observed a large gap of 2,225 cases (8.3%) between “% diagnosed/reported/linked to care” (22,840 cases, 85.6%) and “% retained in care” (20,615 cases, 77.3%). The diagnosis, reporting and linking to care of a newly identified HIV infection or AIDS case occurs according to a well-defined set of procedures in Japan, and this system operates very smoothly. Thus, reporting and linking to care after the diagnosis should logically be 100% in Japan. The gap of 2,225 cases (8.3%) could be a problem in surveillance, i.e., over-estimation of “diagnosed/reported” cases due to double counting, etc. as discussed above. Another possibility is that the large gap is due to a modest counting of patients retained in care. We adopted the total number from the 308 hospitals that replied to all queries (Table 2). We did not include 613 (21,228–20,615) cases reported from 48 (356–308) hospitals since their responses were not complete. Additionally, 26 hospitals (382–356) did not respond to the questionnaire and may have treated HIV-infected patients. Based on a previous survey, presumably more than 500 patients attend these hospitals [35]. Moreover, there are patients who are treated in a few private clinics in Tokyo. Therefore, this gap would decrease through a more extensive survey in the future.

AIDS Core Hospitals were designated initially in 1993 for better HIV/AIDS treatment throughout the country [36]. A few years later, ART was widely introduced (in 1997), and treatment guidelines were updated. Our estimates, such as % on ART/retained in care (91.8% = 18,921/20,615) and treatment success rate (99.1% = 18,756/18,921), seem to be remarkably high and on par with or even better than those reported by Northern European countries or Australia [15,29,37].

Our results suggest that once patients are retained in care, the outcomes of care are then excellent. The high treatment coverage and the trends in the HIV epidemic in Japan are also noteworthy. Since the introduction of ART in 1997, the HIV treatment guidelines have been updated in Japan (concurrently with North America and Europe). The earliest treatment guidelines recommended that treatment is delayed until the CD4+ cell count had fallen below 200 cells/μL [38]. Several treatment guidelines published in 2008 recommended that all individuals with a CD4+ cell count less than 350 cell/μL should be treated. In 2013, WHO recommended starting treatment with a CD4+ cell count less than 500 cell/μL, while in 2015, the START trial suggested that patients with CD4+ cells over 500 cells/μL should be treated. Thus, an extensive scale-up in HIV treatment became the clinical reality rather recently. The numbers of reported cases of both HIV infection and AIDS have plateaued since 2009 and 2013, respectively. High treatment uptake in an AIDS Core Hospital was realized rather recently (over 90% patients on ART since 2013). Presumably, this would also be the case in other AIDS Core Hospitals. Remarkably, high treatment uptake in Denmark (80%) was only attained in approximately 2010 [15]. If Japan maintains a high level of ART coverage and the treatment success rate, we would be in a position to witness the outcome of TasP in the population.

In conclusion, Japan failed to achieve the first two of the three UNAIDS/WHO 90-90-90 targets. To achieve the targets, decreasing the number of undiagnosed PLWHA should be the most important task ahead. If Japan could further improve the quality of the HIV surveillance data, high-level ART implemented in the country would serve to examine the impact of TasP in this nation.

## Supporting information

S1 Questionnaire Questionnaire sent to the AIDS Core Hospitals. (DOCX)

S1 Table Sensitivity analysis of HIV-positive incidence in blood donors. (DOCX)

S2 Table Sensitivity analysis of surveillance data. (DOCX)
