# Supplementary material for: The HIV care cascade: Japanese perspectives
Source: PLoS One. 2017 Mar 20;12(3):e0174360. doi: 10.1371/journal.pone.0174360 (PMC5358866; doi:10.1371/journal.pone.0174360)
Supplement: S1 Questionnaire — (DOCX) [file pone.0174360.s001.docx]

S1 Questionnaire. Questionnaire sent to the AIDS Core Hospitals.

Please indicate the number of patients corresponding to each item.

1. Annual new patients
2. Annual new patients including both treatment-naïve and treated patients.
3. Treatment-naïve patients at first visit among 1-(1).
4. AIDS cases at first visit or admission among 1-(2).
5. Patients with nadir CD4 <200/μL among 1-(2).
6. Regular visitors
7. Regular visitors: visitors during October 1, 2015 – December 31, 2015.
8. Patients with hemophilia among 2-(1).
9. Patients < 15 years/old on December 31, 2015 among 2-(1).
10. Antiretroviral treatment (ART)
11. Patients who initiated or resumed ART during October 1, 2015 – December 31, 2015.
12. Patients on ART.
13. Patients with treatment failure (viral loads >200copis/mL twice successively).
14. Mortality
15. Annual deaths
16. Annual deaths during January 1, 2015 – December 31, 2015.
17. Annual death of patients with hemophilia among 4-(1)-a.
18. Cumulative deaths
19. Cumulative deaths until December 31, 2015.
20. Cumulative death of patients with hemophilia among 4-(2)-a.
21. Hemodialysis
22. Patients on hemo- or peritoneal dialysis.
23. Patients with hemophilia among 5-(1).
