# Supplementary material for: The HIV care cascade: Japanese perspectives
Source: PLoS One. 2017 Mar 20;12(3):e0174360. doi: 10.1371/journal.pone.0174360 (PMC5358866; doi:10.1371/journal.pone.0174360)
Supplement: S1 Table — (DOCX) [file pone.0174360.s002.docx]

**S1 Table. Sensitivity analysis of HIV-positive incidence in blood donors.**

| Age | Sex | Mean first-time donors /year | National age and sex specific population in 2015 | Sensitivity analyses | | | | | |
| --- | --- | --- | --- | --- | --- | --- | --- | --- | --- |
|  |  |  |  | Range of analyses | -20% | -10% | 0 | +10% | +20% |
| 16~19 | M | 79,240 | 2,470,000 | Mean HIV+ /year | 1 | 1 | 1 | 1 | 1 |
|  |  |  |  | Weighted HIV+ number | 31 | 31 | 31 | 31 | 31 |
|  | F | 55,921 | 2,343,000 | Mean HIV+ /year | 0.2 | 0.2 | 0.2 | 0.2 | 0.2 |
|  |  |  |  | Weighted HIV+ number | 8 | 8 | 8 | 8 | 8 |
| 20~24 | M | 68,725 | 3,046,000 | Mean HIV+ /year | 2.2 | 2.8 | 3.4 | 4 | 4.6 |
|  |  |  |  | Weighted HIV+ number | 98 | 124 | 151 | 177 | 204 |
|  | F | 43,491 | 2,922,000 | Mean HIV+ /year | 0.2 | 0.2 | 0.2 | 0.2 | 0.2 |
|  |  |  |  | Weighted HIV+ number | 13 | 13 | 13 | 13 | 13 |
| 25~29 | M | 36,030 | 3,256,000 | Mean HIV+ /year | 5 | 5.6 | 6.2 | 6.8 | 7.4 |
|  |  |  |  | Weighted HIV+ number | 452 | 506 | 560 | 615 | 669 |
|  | F | 18,282 | 3,154,000 | Mean HIV+ /year | 0.4 | 0.4 | 0.4 | 0.4 | 0.4 |
|  |  |  |  | Weighted HIV+ number | 69 | 69 | 69 | 69 | 69 |
| 30~34 | M | 23,510 | 3,685,000 | Mean HIV+ /year | 2.6 | 3.2 | 3.8 | 4.4 | 5 |
|  |  |  |  | Weighted HIV+ number | 408 | 502 | 596 | 690 | 784 |
|  | F | 11,556 | 3,606,000 | Mean HIV+ /year | 0 | 0 | 0 | 0 | 0 |
|  |  |  |  | Weighted HIV+ number | 0 | 0 | 0 | 0 | 0 |
| 35~39 | M | 20,805 | 4,204,000 | Mean HIV+ /year | 2.4 | 2.4 | 2.4 | 2.4 | 2.4 |
|  |  |  |  | Weighted HIV+ number | 485 | 485 | 485 | 485 | 485 |
|  | F | 10,582 | 4,112,000 | Mean HIV+ /year | 0 | 0 | 0 | 0 | 0 |
|  |  |  |  | Weighted HIV+ number | 0 | 0 | 0 | 0 | 0 |
| 40~44 | M | 18,888 | 4,914,000 | Mean HIV+ /year | 2.2 | 2.8 | 3.4 | 4 | 4.6 |
|  |  |  |  | Weighted HIV+ number | 572 | 728 | 885 | 1,041 | 1,197 |
|  | F | 10,840 | 4,818,000 | Mean HIV+ /year | 0 | 0 | 0 | 0 | 0 |
|  |  |  |  | Weighted HIV+ number | 0 | 0 | 0 | 0 | 0 |
| 45~49 | M | 13,233 | 4,355,000 | Mean HIV+ /year | 1.6 | 1.6 | 1.6 | 1.6 | 1.6 |
|  |  |  |  | Weighted HIV+ number | 527 | 527 | 527 | 527 | 527 |
|  | F | 8,820 | 4,308,000 | Mean HIV+ /year | 0 | 0 | 0 | 0 | 0 |
|  |  |  |  | Weighted HIV+ number | 0 | 0 | 0 | 0 | 0 |
| 50~54 | M | 9,187 | 3,968,000 | Mean HIV+ /year | 0.6 | 0.6 | 0.6 | 0.6 | 0.6 |
|  |  |  |  | Weighted HIV+ number | 259 | 259 | 259 | 259 | 259 |
|  | F | 8,051 | 3,962,000 | Mean HIV+ /year | 0 | 0 | 0 | 0 | 0 |
|  |  |  |  | Weighted HIV+ number | 0 | 0 | 0 | 0 | 0 |
| 55~59 | M | 6,072 | 3,730,000 | Mean HIV+ /year | 0.4 | 0.4 | 0.4 | 0.4 | 0.4 |
|  |  |  |  | Weighted HIV+ number | 246 | 246 | 246 | 246 | 246 |
|  | F | 5,440 | 3,786,000 | Mean HIV+ /year | 0 | 0 | 0 | 0 | 0 |
|  |  |  |  | Weighted HIV+ number | 0 | 0 | 0 | 0 | 0 |
| 60~64 | M | 3,925 | 4,151,000 | Mean HIV+ /year | 0 | 0 | 0 | 0 | 0 |
|  |  |  |  | Weighted HIV+ number | 0 | 0 | 0 | 0 | 0 |
|  | F | 3,332 | 4,304,000 | Mean HIV+ /year | 0 | 0 | 0 | 0 | 0 |
|  |  |  |  | Weighted HIV+ number | 0 | 0 | 0 | 0 | 0 |
| 65~69 | M | 287 | 4,660,000 | Mean HIV+ /year | 0 | 0 | 0 | 0 | 0 |
|  |  |  |  | Weighted HIV+ number | 0 | 0 | 0 | 0 | 0 |
|  | F | 228 | 4,984,000 | Mean HIV+ /year | 0 | 0 | 0 | 0 | 0 |
|  |  |  |  | Weighted HIV+ number | 0 | 0 | 0 | 0 | 0 |
| HIV+ first-time blood donors/year | | | | | 18.8 | 21.2 | 23.6 | 26.0 | 28.4 |
| Estimated undiagnosed HIV-positive patients | | | | | 3,168 | 3,498 | 3,830 | 4,161 | 4,492 |
| Estimated diagnosed HIV-positive patients | | | | | 22,840 | 22,840 | 22,840 | 22,840 | 22,840 |
| Estimated PLWHA | | | | | 26,008 | 26,338 | 26,670 | 27,001 | 27,332 |
| % diagnosed patients /PLWHA | | | | | 87.8 | 86.7 | 85.6 | 84.6 | 83.6 |

Mean HIV+ /year: Mean HIV-positive cases in the first-time blood donors between 2011–2015.

Weighted HIV+ number: (Mean HIV+/year) x (National age and sex specific population in 2015) / (Mean first-time donors /year).

The effect of 10-20% difference due to inclusion or exclusion of high-risk groups in the blood donation process was assessed. Ten percent (2.4) or 20% (4.8) of mean HIV+ /year (23.6) were distributed evenly to the four age and sex specific groups with highest HIV+ cases (colored cells).
