# Supplementary material for: The HIV care cascade: Japanese perspectives
Source: PLoS One. 2017 Mar 20;12(3):e0174360. doi: 10.1371/journal.pone.0174360 (PMC5358866; doi:10.1371/journal.pone.0174360)
Supplement: S2 Table — (DOCX) [file pone.0174360.s003.docx]

**S2 Table. Sensitivity analysis of surveillance data.**

| Cumulative report | | Foreigners who left Japan | | Cumulative death | | Undiagnosed patients | Diagnosed patients | PLWHA | % diagnosed patients /PLWHA |
| --- | --- | --- | --- | --- | --- | --- | --- | --- | --- |
|  | s |  | t |  | u | v | w = s – t – u | x = v + w | w / x |
| 100% | 27,434 | 100% | 2,273 | 100% | 2,321 | 3,830 | 22,840 | 26,670 | 85.6% |
| -5% | 26,062 | 100% | 2,273 | 100% | 2,321 | 3,830 | 21,468 | 25,298 | 84.9% |
| -10% | 24,691 | 100% | 2,273 | 100% | 2,321 | 3,830 | 20,097 | 23,927 | 84.0% |
| -20% | 21,947 | 100% | 2,273 | 100% | 2,321 | 3,830 | 17,353 | 21,183 | 81.9% |
| 100% | 27,434 | -20% | 1,818 | 100% | 2,321 | 3,830 | 23,295 | 27,125 | 85.9% |
| 100% | 27,434 | -10% | 2,046 | 100% | 2,321 | 3,830 | 23,067 | 26,897 | 85.8% |
| 100% | 27,434 | -5% | 2,159 | 100% | 2,321 | 3,830 | 22,954 | 26,784 | 85.7% |
| 100% | 27,434 | 100% | 2,273 | 100% | 2,321 | 3,830 | 22,840 | 26,670 | 85.6% |
| 100% | 27,434 | 5% | 2,387 | 100% | 2,321 | 3,830 | 22,726 | 26,556 | 85.6% |
| 100% | 27,434 | 10% | 2,500 | 100% | 2,321 | 3,830 | 22,613 | 26,443 | 85.5% |
| 100% | 27,434 | 20% | 2,728 | 100% | 2,321 | 3,830 | 22,385 | 26,215 | 85.4% |
| 100% | 27,434 | 100% | 2,273 | 5% | 2,437 | 3,830 | 22,724 | 26,554 | 85.6% |
| 100% | 27,434 | 100% | 2,273 | 10% | 2,553 | 3,830 | 22,608 | 26,438 | 85.5% |
| 100% | 27,434 | 100% | 2,273 | 20% | 2,785 | 3,830 | 22,376 | 26,206 | 85.4% |
| 100% | 27,434 | 10% | 2,500 | 10% | 2,553 | 3,830 | 22,381 | 26,211 | 85.4% |
